# Corrigendum to ‘The battle against fungi lessons in antifungal stewardship from COVID 19 times’ [International Journal of Antimicrobial Agents Volume 62/1 (2023) 106846]^⁎^

**DOI:** 10.1016/j.ijantimicag.2023.106901

**Published:** 2023-08

**Authors:** Souha S. Kanj, Sara F. Haddad, Jacques F. Meis, Paul E. Verweij, Andreas Voss, Riina Rautemaa-Richardson, Gabriel Levy-Hara, Anuradha Chowdhary, Abdul Ghafur, Roger Brüggemann, Abhijit M. Bal, Jeroen Schouten

**Affiliations:** a Division of Infectious Diseases, Internal Medicine Department, American University of Beirut Medical Center, Beirut, 1107 2020, Lebanon; b Department of Medical Microbiology and Infectious Diseases, Canisius-Wilhelmina Hospital, 6532 SZ Nijmegen, The Netherlands; Centre of Expertise in Mycology Radboudumc, Nijmegen, The Netherlands; c Department of Medical Microbiology and Radboudumc Center for Infectious Diseases; Centre of Expertise in Mycology Radboudumc/Canisius-Wilhelmina Hospital, Radboud University Medical Center, 6500 HB, Nijmegen, The Netherlands; d Department of Medical Microbiology and Infection prevention, University Medical Center Groningen, The Netherlands; e Mycology Reference Centre Manchester and Department of Infectious Diseases, Wythenshawe Hospital, Manchester University NHS Foundation Trust; and Division of Evolution, Infection and Genomics, Faculty of Biology, Medicine and Health, University of Manchester, UK; f Infectious Diseases Unit, Hospital Carlos G. Durand, Av Díaz Vélez 5044, 1416, Buenos Aires, Argentina; g Medical Mycology Unit, Department of Microbiology, Vallabhbhai Patel Chest Institute, University of Delhi, Delhi, India; National Reference Laboratory for Antimicrobial Resistance in Fungal Pathogens, University of Delhi, Delhi, 110007, India; h Department of Infectious Diseases and Clinical Microbiology, Apollo Cancer Institute, Chennai, Tamil Nadu, India; i Department of Pharmacy, Centre of Expertise in Mycology Radboudumc/Canisius-Wilhelmina Hospital, Radboud University Medical Center, Nijmegen, The Netherlands; j Department of Microbiology, Queen Elizabeth University Hospital, Glasgow, Scotland, United Kingdom; k Department of Intensive Care and Radboudumc Center for Infectious Diseases, Radboud University Medical Center, Nijmegen, The Netherlands

The authors regret that the original article “**The battle against fungi lessons in antifungal stewradship from COVID 19 times” was incorrect. The correct title is “The battle against fungi: lessons in antifungal stewardship from COVID 19 times”**.

The authors would like to apologise for any inconvenience caused.

